# Serum Calprotectin: A Potential Biomarker for Neonatal Sepsis

**DOI:** 10.1155/2015/147973

**Published:** 2015-08-26

**Authors:** Lidia Decembrino, Mara De Amici, Margherita Pozzi, Annalisa De Silvestri, Mauro Stronati

**Affiliations:** ^1^Neonatology and Neonatal Intensive Care Unit, IRCCS San Matteo Foundation, 27100 Pavia, Italy; ^2^Pediatric Clinic, IRCCS San Matteo Foundation, 27100 Pavia, Italy; ^3^Biometry and Statistics Service, IRCCS San Matteo Foundation, 27100 Pavia, Italy

## Abstract

*Introduction. *The correct diagnosis of neonatal sepsis is a relevant problem because sepsis is one of the most important causes of neonatal morbidity, mortality, and prolonged hospital stay. Calprotectin is an antimicrobial, calcium and zinc binding heterocomplex protein that could be used as a nonspecific marker for activation of granulocytes and mononuclear phagocytes. Calprotectin has been proposed for the diagnosis of inflammatory conditions. Our aim is to study serum calprotectin as a biomarker for neonatal sepsis diagnosis. *Methods.* 41 (20 females, 21 males) infants who underwent blood culture due to suspected sepsis were enrolled in the study. Serum calprotectin was measured by a commercial ELISA assay (Calprest, Eurospital, Trieste, Italy). Statistical analysis was performed using the statistical software package Stata 13.1 (Stata Corporation, College Station, Texas, USA). *Results.* 8 neonates (19.51%) showed sepsis with positive culture and 33 (80.49%) showed suspected sepsis. The optimal cut-off for calprotectin is 2.2 *μ*g/mL with a sensitivity of 62.5% and a specificity of 69.7%. *Conclusions.* Calprotectin may be considered a promising early, sensitive, specific marker of sepsis thanks to the importance of calprotectin in defense mechanisms and physiological functions of the immune system.

## 1. Introduction

Sepsis is one of the most important causes of neonatal morbidity, mortality, and prolonged hospital stay, particularly [1, 2] in preterms, due to their immature immune response and exposure to infectious risks in neonatal intensive care units (NICUs). In this contest, an early diagnosis becomes crucial as signs of sepsis in the newborn are nonspecific [1–3].

Blood culture remains the gold standard test for neonatal sepsis; even though the result is not available before 24–48 h and there are possible false negative responses in many instances. For this reason, a broad spectrum of inflammatory markers has been proposed for the diagnosis of neonatal sepsis [4]. Calprotectin is an antimicrobial, calcium and zinc binding heterocomplex protein contained in the cytosol fraction of innate immunity cells and released immediately after host-pathogen interaction. For this it has been used as a nonspecific marker for activation of granulocytes and mononuclear phagocytes. [5, 6] In addition to protecting cells against microorganisms, calprotectin regulates adhesion of leukocytes to the endothelium and extracellular matrix during the inflammatory process [4, 7–9]. So calprotectin has been proposed for the diagnosis of inflammatory conditions.

While in young infants and healthy preterm and term babies high faecal calprotectin concentrations are considered normal [10–15], in VLBW infants developing severe abdominal disease like necrotizing enterocolitis (NEC), faecal calprotectin tends to increase and it has been used as a marker for early diagnosis [14–16]. Calprotectin in the CSF was revealed to be less efficient marker than Lactoferrin in distinguishing between bacterial and viral meningitis [17]. Its use in the diagnosis of neonatal sepsis based on its blood measurement remains unexplored. Terrin et al. [18] investigated the diagnostic role of serum calprotectin (SC) in VLBW infants, considering that serum calprotectin could be a practical and accurate marker in the diagnostic approach to VLBW infants with suspected sepsis. Serum calprotectin levels revealed a sensitive diagnostic marker for late onset neonatal sepsis with a positive correlation with blood cultures, poor outcomes, and mortality [19].

## 2. Materials and Methods

All infants who were admitted to our Department between September 2011 and October 2012 and underwent blood culture due to suspected sepsis were enrolled in the study. All fulfilled at least one of the following clinical signs and symptoms: fever or hypothermia, respiratory distress including cyanosis and apnea, feeding difficulties, lethargy or irritability, hypotonia, seizures, bulging fontanel, poor perfusion, bleeding signs, abdominal distention, hepatomegaly, bloody stools, and unexplained jaundice. White blood cell (WBC) count, neutrophil, platelet count, and serum C-reactive protein (CRP) were used as other infection markers [1, 20]. Serum calprotectin was measured by a commercial ELISA assay (Calprest, Eurospital, Trieste, Italy). Continuous variables were expressed as mean and standard deviation (SD). The optimal cut-off of infection markers value to distinguish septic from nonseptic patients among subjects investigated for suspected sepsis was determined using receiver operating characteristic (ROC) curve analysis. Sensitivity, specificity, and likelihood ratio (LR) are reported. Diagnostic performance was evaluated with area under ROC curve (AUC), reported with 95% confidence interval (CI). AUC of each marker was compared with AUC of calprotectin (against the same gold standard) by means of a nonparametric approach [21].

A sample of 8 from the positive group and 40 from the negative group achieves 80% power to detect a difference of 0.2500 between a diagnostic test with an area under the ROC curve (AUC) of 0.8500 and another diagnostic test with an AUC of 0.6000. The ratio of the standard deviation of the responses in the negative group to the standard deviation of the responses in the positive group for diagnostic test 1 is 1.000 and for diagnostic test 2 is 1.000. The correlation between the two diagnostic tests is assumed to be 0.600 for the positive group and 0.600 for the negative group.

## 3. Results

**Figure 1:**
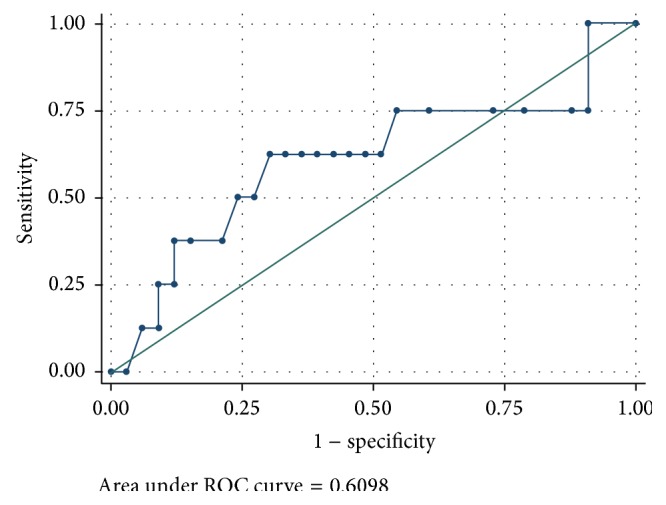
Calprotectin: ROC curve.

41 infants were enrolled (20 F/21 M); BW was 2171 g (SD 984). 8/41 neonates (19.51%) (3 F/5 M) showed sepsis with positive culture (by* Klebsiella*,* Streptococcus*,* Staphylococcus*, or* P. aeruginosa*). 33/41 neonates showed suspected sepsis.  Table 1 shows the mean and standard deviation of infection markers used together with AUC and relative 95% CI (Table 1). Without presenting a very high value of AUC, the calprotectin has a value comparable to those of CRP and platelets (Figure 1). The optimal cut-off is 2.2 (sensitivity and specificity: 62.5% and 69.7%, resp.; LR+: 2.06, LR−: 0.53).

## 4. Discussion

Prompt diagnosis of sepsis is crucial to establishing treatment and modifying neonatal prognosis. An ideal marker for sepsis should guarantee an early diagnosis, high specificity and sensitivity, a correlation with the severity of the disease, a prognostic value, and a differential diagnosis between infectious and noninfectious etiologies and should be easily detectable.

First of all, for an early diagnosis, the presence of subtle or clear signs of sepsis requires immediate laboratory investigation. Various laboratory tests can provide diagnostic information such as abnormal values of white blood cells < 4000 or > 25000/mL and platelet count ≤ 150,000/mm^3^ which can shrink a few hours or a few days before beginning of a clinical sepsis.

One of the most commonly used markers for the diagnosis of neonatal sepsis is the C-reactive protein (CRP); however, CRP concentration increases rather slowly in the initial phase of the inflammatory response to pathogens, and its sensitivity is insufficient. Furthermore, elevated levels of CRP may be more difficult to interpret, because factors such as premature rupture of membranes (PROM), maternal fever, pregnancy-induced hypertension, prenatal steroid use, fetal distress, and gestational age may influence its kinetics. Additionally, a physiologic variation limits its use in the first days of life. The specificity and sensitivity of procalcitonin (PCT) are relatively low. Its levels increase more rapidly and may be more useful for detection of EOS as compared with CRP but it may also increase with noninfectious causes such as respiratory distress syndrome, trauma, and major and cardiac surgery and physiologically during the first 24 hours of birth. The usefulness of neutrophil CD64 (nCD64) is related to high negative predictive value but there is scarcity of medical evidence to recommend nCD64 for routine evaluation of neonatal infection. Blood concentration of cytokines is not clear in concert with the course of sepsis, making their interpretation difficult [22].

Our study was performed to evaluate if serum calprotecin can be used as a marker for early diagnosis of sepsis. The data obtained show that the plasma concentration of calprotectin is increased in infants with sepsis. The plasma levels of calprotectin do not seem to be influenced by age, sex, age at enrollment, mode of delivery, or white blood cell count in small blood samples. The preliminary results are encouraging and seem to indicate an early CRP plasmatic level early and before the CRP increase. An optimal cut-off value of 2.2 *μ*g/mL plasmatic calprotectin was identified to distinguish between infants with sepsis and infants without sepsis, with sensitivity and specificity of 62.5% and 69.7%, respectively, whereas CRP for a cut-off of 0.6 showed a sensitivity of 50% and specificity of 66.7%.

## 5. Conclusion

Even though the diagnosis of sepsis cannot be based on biological markers and actually blood culture remains the gold standard for diagnosis, our experience reveals that calprotectin may be considered a promising early, sensitive, specific marker for sepsis thanks to the importance of calprotectin in defense mechanisms and physiological functions of the immune system.

## Figures and Tables

**Table 1:** Mean values, SD, AUC, and 95% CI of markers of infection.

	Negative culture Mean values/SD	Positive culture Mean values/SD	AUC/IC	*P* AUC versus calprotectin
WBC (10^3^/mm^3^)	13.0 (4.4)	13.3 (6.5)	0.47 (0.14–0.80)	0.21
Neutrophils (10^3^/mm^3^)	6.4 (3.9)	5.8 (5.1)	0.42 (0.13–0.70)	0.08
Platelets (10^3^/mm^3^)	258 (99)	414 (305)	0.66 (0.37–0.96)	0.63
RCP (mg/dL)	1.5 (2.9)	6.0 (9.4)	0.70 (0.46–0.95)	0.53
Calprotectin	1.9 (1.9)	2.7 (2.3)	0.61 (0.35–0.87)	—
